# Moderate Drinking and Reduced Risk of Heart Disease

**Published:** 1999

**Authors:** Arthur L. Klatsky

**Affiliations:** Arthur L. Klatsky, M.D., is a senior consultant in cardiology at the Division of Cardiology, Department of Medicine, and the Division of Research, Kaiser Permanente Medical Center, Oakland, California

**Keywords:** moderate AOD use, heavy AOD use, coronary artery disorder, risk factors, protective factors, AODR (alcohol and other drug related) disorder, AODR mortality, alcoholic cardiomyopathy, hypertensive disorder, cardiac arrhythmia, stroke, alcoholic beverage, public health, AOD use frequency, literature review

## Abstract

Although heavier drinkers are at increased risk for some heart diseases, moderate drinkers are at lower risk for the most common form of heart disease, coronary artery disease (CAD) than are either heavier drinkers or abstainers. This association has been demonstrated in large-scale epidemiological studies from many countries. Abstainers may share traits potentially related to CAD risk, such as psychological characteristics, dietary habits, and physical exercise patterns. However, evidence supports a direct protective effect of alcohol, even after data have been adjusted for the presence of these factors. The alcohol-CAD relationship is also independent of the hypothetically increased risk status among abstainers who stopped drinking for medical reasons. All alcoholic beverages protect against CAD, although some additional protection may be attributable to personal traits or drinking patterns among people who share some beverage preferences or to nonalcohol ingredients in specific beverages. Alcohol’s protective effect may result from favorable alterations in blood chemistry and the prevention of clot formation in arteries that deliver blood to the heart muscle. Because CAD accounts for a large proportion of total mortality, the risk of death from all causes is slightly lower among moderate drinkers than among abstainers, but heavier drinkers are at considerably higher total mortality risk.

Diseases of the heart and blood vessels (i.e., cardiovascular diseases [CVDs]) are a major cause of illness and disability in the United States. Heart disease is the leading cause and stroke is the third leading cause of death among adult Americans. Together, these two conditions account for more than 40 percent of all deaths annually (Dufour 1996).

Researchers have studied extensively the role of alcohol in CVD, especially in relation to drinking level. Heavier drinking (see the following section, “ Definitions of Moderate Drinking” ) is related to higher risk of heart muscle disorders (i.e., cardiomyopathy), high blood pressure (i.e., hypertension), brain damage from ruptured blood vessels (i.e., hemorrhagic stroke), and heart rhythm irregularities (i.e., arrhythmias). Lighter drinking is related to lower risk of coronary artery disease (CAD) and of ischemic stroke, which is characterized by blockage of blood vessels that supply the brain.^1^

Sweeping generalizations circulated in the popular media have perpetuated public misconceptions about the effects of moderate drinking on CVD. In truth, the relationships between drinking and CVD are both complex and interconnected (Klatsky 1995*a*). Discussions of alcohol’s effects on CVD must clearly differentiate between different levels of drinking as well as the specific type of CVD being considered.

This article briefly reviews the effects of heavier drinking on certain CVDs and considers at greater length the cardiovascular effects of moderate drinking. The article concentrates on CAD for two major reasons. First, CAD is the most common type of CVD and therefore dominates epidemiological statistics when CVDs are studied as a group. Second, alcohol has been reputed to have a protective effect against CAD. The discussion of CAD suggests possible mechanisms to account for this protective effect, including the potential role of beverage type. Finally, the article discusses implications of the alcohol-CVD relationship in terms of total mortality and overall public health.

## Definitions of Moderate Drinking

Definitions of moderate drinking vary widely (see the article by Dufour, pp. 5–14). This article defines moderate drinking as the consumption of fewer than three standard drinks per day. A standard drink is equivalent to approximately 12 ounces (oz) of beer, 5 oz of wine, or 1.5 oz of distilled spirits, each of which contains approximately 12 grams (0.5 oz) of alcohol.

Problems inherent to epidemiological studies of alcohol and CVD include individual susceptibility and the categorization of drinking levels. Individual factors that influence interpretation of study results include sex, age, dietary habits, cigarette smoking, the consumption of caffeinated beverages, and various psychosocial factors that are difficult to characterize and measure.

The characterization of drinking levels is complicated by the use of subjects’ self-reported estimates. In particular, heavier drinkers may underestimate their alcohol consumption. If some proportion of heavy drinkers report lighter drinking, then the “ lighter” drinking group will include some people who really drink more, and a condition related only to heavy drinking (e.g., alcoholic cardiomyopathy) may erroneously appear to be related to moderate alcohol consumption (Klatsky 1994).

Another potential source of error is failure to consider individual differences in drinking patterns. Subjects in population studies are generally requested to describe their average total alcohol consumption over a given time period (e.g., 1 week or 1 month). Thus, a person who habitually consumes 2 drinks each evening might report the same average weekly consumption as a person who hypothetically consumes 14 drinks within a few hours every Saturday night. The health risks posed by such widely varying patterns of consumption may differ substantially.

## Effects of Heavy Drinking

### Alcoholic Cardiomyopathy

Clinicians and researchers have long recognized that alcohol consumption can directly damage heart muscle cells independently of any other cardiovascular effect (Klatsky 1995*a*). Breathlessness and fatigue may be early signs of such heart muscle disease (i.e., cardiomyopathy). Complications may develop as the disease progresses, including heart failure, embolism, and arrhythmias, possibly resulting in sudden death.

Although cardiomyopathies can be caused by viral infection, exposure to toxic substances and, possibly, genetic factors, most cases are considered to be of unknown cause. The type of heart muscle damage produced by alcohol is called *dilated* cardiomyopathy, because one or more heart chambers are abnormally distended with blood. Alcoholic and nonalcoholic cardiomyopathy are not readily distinguishable either clinically or pathologically. Thus, alcohol cardiomyopathy is generally diagnosed when dilated cardiomyopathy of unknown origin is encountered in a patient with a history of long-term heavy drinking. The proportion of cardiomyopathy attributable to alcohol is unknown.

Although substantial circumstantial evidence implicates alcohol as a cause of dilated cardiomyopathy, the absence of specific diagnostic tests seriously impedes epidemiological study. The most convincing circumstantial evidence for alcoholic cardiomyopathy is the extensive data, in animals and humans, of nonspecific cardiac abnormalities related to alcohol. One key report (Urbano-Marquez et al. 1989) showed a clear relationship in alcoholics between lifetime alcohol consumption and both structural and functional abnormalities of heart and skeletal muscle. The amounts of alcohol necessary to produce such evidence were large, equivalent to eight or more drinks per day over a period of 20 years. Thus, moderate drinking does not appear to increase the risk of alcoholic cardiomyopathy.

Both clinical observations and experimental data (Ballester et al. 1997) suggest that alcoholic cardiomyopathy can improve with abstinence, which is therefore a cornerstone of therapy.

### Hypertension

Although an association between heavy alcohol consumption and hypertension was recognized as early as 1915 (Lian 1915), the epidemiological significance of this finding was largely ignored for the next 60 years. Since the mid-1970s, alcohol has joined obesity and salt intake as an acknowledged factor potentially contributing to the risk for hypertension. Two types of epidemiological studies provide evidence for this relationship. Cross-sectional studies seek a correlation between reported alcohol use and blood pressure; prospective studies follow subjects over time to document the development of increased blood pressure in relation to reported alcohol use. Of the more than 50 cross-sectional and 10 prospective population studies, almost all have demonstrated a link between alcohol and hypertension in nonhospitalized persons in a number of countries (Klatsky 1995*b*). Studies differ about whether a threshold of alcohol consumption exists below which the hypertensive effect does not occur. The preponderance of evidence suggests that clinically significant hypertension is related only to heavier drinking.

Two Kaiser Permanente studies are among the largest of the cross-sectional population surveys.

The first of these studies (Klatsky et al. 1977) showed slightly lower blood pressures among women who consumed no more than two drinks per day; the study found no such relationship among moderately drinking men, although blood pressures were highest among heavier drinkers (three or more drinks per day) in both sexes. These results were independent of other potentially contributory factors such as age, sex, race, smoking, coffee intake, reported past heavy drinking, education, obesity, and habitual salt use. Hypertension (e.g., blood pressure greater than 160/95 mm Hg [millimeters of mercury], the units of measure on a blood pressure reading) occurred among twice as many people who reported consuming six or more drinks per day as among light drinkers or abstainers.

The second Kaiser Permanente study (Klatsky et al. 1986*a*) demonstrated similar results following adjustment for the influence of age, obesity, smoking, consumption of coffee or tea, and certain biochemical factors measured by blood chemistry analysis. Former drinkers did not have higher blood pressure than lifelong abstainers, and subjects’ reported alcohol consumption during the week before examination suggested rapid reversal of alcohol-associated hypertension with abstinence.

Several clinical experiments have been undertaken on alcohol’s short-term blood pressure effects (i.e., effects that develop within a period of a few days to several weeks). Consumption of three to eight alcoholic drinks per day was associated with increased blood pressure, which decreased upon abstention or marked reduction of alcohol consumption (Klatsky 1995*b*). The association between alcohol consumption and blood pressure appears to be independent of salt intake, physical activity, and psychosocial stress. No elevations of blood pressure have been reported as a result from alcohol withdrawal (Klatsky 1995*b*).

These results suggest that alcohol plays a contributory role in the development of hypertension, at least in the short term. However, research has failed to provide a convincing biological explanation for alcohol’s putative causal effect. Studies of the long-term health effects of sustained hypertension (e.g., CAD, stroke, and congestive heart failure) are difficult to interpret because of alcohol’s ability to influence these same conditions directly. Estimates of the proportion of all hypertension attributable to alcohol consumption range from 5 to 30 percent. Because hypertension affects an estimated 50 million Americans, even the lowest estimate suggests that heavy drinking may be the most common cause of potentially reversible hypertension in developed societies (Fifth Report 1993).

### Cardiac Arrhythmias

An association of heavier alcohol consumption with disturbances of normal heart rhythm has been suspected for decades. Ettinger and colleagues (1978), describing a series of patients hospitalized for episodes of arrhythmia following heavy weekend or holiday drinking sprees, referred to this phenomenon as the “ holiday heart syndrome.” Symptoms of acute alcohol-induced arrhythmia include palpitations and difficulty breathing. Onset usually follows a large meal accompanied by heavy drinking. Symptoms usually abate on abstinence, with or without other specific treatment.

A study of approximately 4,000 subjects investigated the occurrence of various arrhythmias, including rapid, irregular, and premature contractions of the upper chambers of the heart. The relative risk for these conditions (Cohen et al. 1988) was at least twice as high among people consuming six or more drinks per day as among people consuming less than one drink per day. Certain arrhythmias are extremely common in older persons, and only a small proportion of arrhythmias result from heavy alcohol consumption.

### Hemorrhagic Stroke

Alcohol-stroke relationships are complex (Van Gign et al. 1993). Several reports suggest that alcohol consumption, especially at heavier drinking levels, is associated with higher risk of stroke. Some studies examined only drinking sprees; others did not differentiate between hemorrhagic and ischemic strokes. Several studies have suggested that alcohol is related only to hemorrhagic stroke; others have shown drinkers to be at *higher* risk for hemorrhagic stroke but at *lower* risk for ischemic stroke. Elevated blood pressure associated with heavier drinking may contribute to the relationship between heavy alcohol consumption and hemorrhagic stroke, and alcohol’s inhibitory effects on the mechanism of blood clotting may contribute to increased risk at both lighter and heavier drinking levels. Hemorrhagic strokes carry a high risk of death (as much as 50 percent in several reports) and permanent disability. No clear data exist that afford an estimate of the proportion of such deaths that might be attributable to alcohol.

## Effects of Moderate Drinking

### Ischemic Stroke

Although hemorrhagic strokes are associated with higher mortality, ischemic strokes are a common cause of serious disability, especially among the elderly (see glossary, p. 23). The relationship of alcohol to ischemic stroke is complex, because underlying heart disease of almost any type predisposes to embolism, as does atherosclerotic disease in the blood vessels within, or leading to, the brain. Although further study is needed, the preponderance of evidence suggests that moderate drinking is associated with lower risk of ischemic strokes (Van Gign et al. 1993).

### Coronary Artery Disease

The most common heart disease category is CAD, which causes about 60 percent of CVD deaths and 25 percent of *all* deaths (Dufour 1996; Klatsky et al. 1990*a*, 1992). A common manifestation of CAD is angina pectoris, characterized by chest pain or discomfort, often of a vague or nonspecific nature. The symptoms of angina were described more than two centuries ago by Heberden (1786), who also noted that “ wine and spiritous liquors . . . afford considerable relief.” Alcohol was widely presumed to alleviate angina by dilating the coronary blood vessels. Electrocardiographic measurements taken from patients during exercise, however, suggest that alcohol does not improve coronary blood flow or increase oxygen delivery to heart muscle. Thus, the perceived beneficial effects of alcohol consumption may be subjective, perhaps resulting from alcohol’s sedating or anesthetic effects. Rather than being beneficial, such effects may dangerously mislead the patient by masking symptoms of serious heart disease (Klatsky 1994).

Early in this century, some researchers reported that increasing levels of alcohol consumption were associated with *decreased* prevalence of atherosclerotic disease, including CAD. One explanation offered was that premature deaths related to heavy alcohol consumption precluded the gradual development of chronic age-related changes that lead to CAD. Research since 1974 (Klatsky 1994) has confirmed this relationship in various countries, several racial groups, and both sexes for both fatal and nonfatal CAD.

#### Epidemiological Evidence

Epidemiological investigations into the relationship between alcohol consumption and CAD include international comparisons, time-trend analyses, case control studies, and longitudinal population studies. Because the symptoms of angina are subjective and variable, studies have concentrated on objective, readily defined events, such as CAD-related hospitalization or death.

Most studies show that heavier drinkers are at similar or lower risk of hospitalization for CAD than are moderate drinkers. Several population studies show a progressive inverse relationship between CAD-related deaths and the amount of alcohol consumption; that is, the rate of CAD deaths declines steadily with increasing alcohol intake. Data from other studies, however, indicate that lighter drinkers are at lower risk for CAD deaths than are either heavier drinkers or abstainers. This type of association can be depicted graphically by a U-shaped curve, with the lowest risk among people who consume moderate amounts of alcohol, specifically one to three drinks per day.

Some controversy persists about the apparent higher CAD risk among abstainers. For example, a much publicized hypothesis (Shaper et al. 1988) suggests that the group of abstainers studied may have included persons with pre-existing alcohol-related health problems who were already at high CAD risk (i.e., “sick quitters” ). However, studies that differentiate between lifelong abstainers and past drinkers suggest that *both* of these types of nondrinkers are at higher risk of CAD than are current drinkers (Renaud et al. 1993; Maclure 1993; Klatsky 1994). In addition, a prospective Kaiser Permanente study of alcohol habits and CAD hospitalizations (Klatsky et al. 1986*b*) showed that former drinkers and infrequent drinkers (i.e., those who consumed less than one drink per month) were at similar CAD risk as lifelong abstainers. All other subjects had a lower CAD risk independent of a number of potential indirect explanations (i.e., confounders), including beverage choice (see the section, “ The Role of Beverage Choice,” p. 19) and baseline CAD risk (i.e., pre-existing CAD risk as evaluated at the patient’s initial examination).

In a prospective study of total CVD mortality (Klatsky et al. 1990*a*), former drinkers had higher age-adjusted CAD and overall CVD mortality risk than lifelong abstainers, but the difference disappeared when adjusted for other traits. Among current drinkers, lighter drinkers had the lowest risk for both total CVD deaths and CAD deaths, yielding U-shaped mortality curves with lowest risk at one to two drinks per day and at three to five drinks per day, respectively. Mortality curves were not influenced by differences in patients’ baseline CAD risk or by the occurrence of pre-existing CAD. Results of the study also confirmed the observation that alcohol’s CVD effects depend on the specific condition (see table 1).

The Nurses Health Study (Stampfer et al. 1988), a large prospective study of women free of baseline CAD, showed a progressive inverse relationship of alcohol use to major CAD, independent of any previous reduction in alcohol consumption or nutrient intake. For women who reported daily alcohol intake of approximately two drinks per day, their relative risk^2^ of CAD was 0.4. The *overall* net beneficial effects of moderate alcohol in these women was present only among persons at above-average CAD risk (basically, age 50 or older); in other women adverse effects of alcohol dominated or canceled the benefit (Fuchs et al. 1995).

Other large prospective studies also confirm the lower CAD risk of drinkers, independent of confounders or the occurrence of other diseases. The American Cancer Society Study, a 9-year prospective mortality study of 490,000 men and women, showed a 30- to 40-percent lower total CVD mortality among both men and women who drink one or two drinks per day (Thun et al. 1997). In the Health Professional Followup Study of 51,529 men (Rimm et al. 1991) who were well controlled for dietary habits, newly diagnosed CAD was inversely related to increasing alcohol intake. A study of both men and women, the Auckland Heart Study (Jackson et al. 1991), was designed to study the hypothesis that persons at high CAD risk were likely to become nondrinkers; the analysis showed that moderate drinkers had lower CAD risk than both lifelong abstainers and former drinkers.

Reduced risk of CAD presents at various ages, although its impact on total mortality in Kaiser Permanente studies was clearest in older age brackets, and the adverse effects of alcohol were greater among younger persons. Among persons older than age 60, overt or latent CAD may play a role in risk of death from causes other than CAD (Klatsky et al. 1992).

#### Possible Protective Mechanisms

Possible mechanisms exist by which alcohol consumption might protect against CAD (Renaud et al. 1993; Klatsky 1994). The most well-established protective effect of alcohol consumption is the increased concentration of a natural component of human blood called high-density lipoprotein (HDL) cholesterol. This substance, often referred to in the popular media as the “ good cholesterol,” may protect against CAD by helping to remove fatty deposits from within large blood vessels. In the absence of severe liver impairment, alcohol ingestion raises HDL levels.^3^ Subtypes of HDL exist, and some data suggest that the HDL_2_ subtype, which is less related to alcohol consumption, may be more protective than other HDL subtypes. More recent studies, however, suggest that both HDL_2_ and HDL_3_ are protective. Biochemical pathways for the HDL effect of alcohol are poorly understood (Gaziano et al. 1993; Renaud et al. 1993; Klatsky 1994).

Four studies have examined the hypothesis that alcohol consumption protects against CAD by increasing HDL cholesterol levels (Criqui et al. 1987; Suh et al. 1992; Gaziano et al. 1993; Marques-Vidal et al. 1996). Findings suggest that higher HDL levels in drinkers mediate approximately one-half of the lower CAD risk. One report (Gaziano et al. 1993) suggests that both HDL_2_ and HDL_3_ are involved in this protective effect.

Some data suggest that alcohol affects various aspects of blood clotting (Renaud et al. 1993; Klatsky 1994; Ridker et al. 1994). Such an action of alcohol could partially account for the lower CAD risk at very light drinking levels (e.g., several drinks per week) seen in several of the epidemiologic studies, but this protective mechanism is less established than the HDL cholesterol pathway.

#### The Role of Beverage Choice

In the early 19th century, a perceptive Irish physician with a great interest in angina pectoris described what has come to be known as the “ French Paradox” (Black 1819). Noting the high prevalence of angina in Ireland compared with that in France, he attributed the difference to “the French habits and modes of living, coinciding with the benignity of their climate and the peculiar character of their moral affections.” Using observational evidence arguably less circumstantial, researchers are still striving 180 years later to define those favorable “ habits and modes of living.”

Over the past two decades, international comparison studies (St. Leger et al. 1979; Rimm et al. 1996) have documented a lower prevalence of CAD in wine-drinking countries than in beer- or liquor-drinking countries. These findings are complemented by the presence in wine of potentially beneficial nonalcoholic substances.^4^ These substances appear to fall into three categories. The first group consists of chemicals that can inhibit blood clotting. A second group consists of chemicals that can relax the walls of blood vessels, potentially dilating constricted coronary arteries. Finally, certain chemicals called polyphenols can interfere with the metabolic process by which LDL cholesterol promotes fat deposition within blood vessels. The mechanism of this effect appears to be based on the polyphenols’ antioxidant properties. However, although diets high in antioxidants seem protective against CAD, it should be noted that prospective clinical trials of antioxidant supplements remain inconclusive (Klatsky et al. 1997).

Prospective population studies provide no consensus about the role of beverage choice in alcohol-related CAD risk. Those studies with relevant data show that statistically significant inverse relationships to CAD for beer, liquor, and wine were generally accompanied by inverse, nonsignificant relationships for other alcoholic beverage types (Renaud et al. 1993; Klatsky 1995*a*; Rimm et al. 1996).

A Kaiser Permanente study determined the CAD risk per drink per day associated with each type of alcoholic beverage among 3,931 patients hospitalized for CAD (Klatsky et al. 1997). Results, *not* controlled for total alcohol intake, showed inverse relationships between daily CAD risk and all alcoholic beverage types. When results were adjusted to account for total alcohol consumption, only beer use by male subjects remained significantly related to daily CAD risk (see table 2). CAD risk did not differ significantly among subjects who drank red wine, white wine, both red and white wine, or other types of wine. These researchers concluded that (1) all alcoholic beverage types protect against CAD and (2) some additional protection may be attributable to personal traits (e.g., race and gender) or drinking patterns (e.g., number of drinks per day) that may characterize groups of people who share certain beverage preferences. Similarly, earlier studies had suggested that the decreased risk of death from CAD among drinkers who prefer wine might be related to group differences in dietary habits, physical exercise, and the use of antioxidant supplements (Klatsky and Armstrong 1993; Klatsky et al. 1990*b*).

The hypothesis that wine drinkers are at lower CAD risk than persons drinking spirits or beer is supported by the Copenhagen City Heart Study (Grønbæk et al. 1995). Risk of CAD showed less of a decrease among beer drinkers, and it increased among drinkers of spirits compared with nondrinkers. In addition, the wine drinkers showed a lower risk of non-CVD mortality at levels of wine consumption up to an average of three to five drinks per day. Interestingly, wine drinkers who participated in this study reported a more healthy diet than did drinkers of beer or spirits (Tjønneland et al. 1999; Klatsky 1999). Thus, it appears that the healthier lifestyle of wine drinkers is not limited to those in the United States.

Although the effects of alcoholic beverage choice on CAD risk remain controversial, most researchers concluded that available evidence does not support a *major* role for beverage choice (Rimm et al. 1996) and that no compelling health-related data exist that preclude personal preference as a guide to one’s choice of beverage.

#### Does Moderate Drinking Decrease CAD Risk?

The validity of the inverse alcohol-CAD relation is supported by the following evidence discussed in this section:

- Independence of results from subjects’ CAD risk at baseline examination
- Absence of higher CAD risk among subjects who had reduced their alcohol consumption for medical reasons
- Evidence that the higher unadjusted CAD risk of former drinkers is attributable to confounding factors associated with past alcohol use
- Absence of an association between CAD risk and maximal past alcohol consumption among former drinkers
- Absence of a relationship between infrequent alcohol consumption (i.e., less than one drink per month) and CAD risk
- Similar reduction of CAD risk regardless of specific beverage preference.

An appropriately designed, prospective-controlled clinical trial could definitively rule out indirect explanations for an inverse alcohol-CAD association. If many medical conditions appeared to be related inversely to lighter drinking, the alcohol-CAD relationship would be less convincing; however, the relative *specificity* of the association is strong evidence of alcohol’s direct protective effect. CAD stands almost alone in its inverse relationship to lighter drinking. Thus, nondrinkers’ increased CAD risk most likely does not represent a general predilection for serious illness.

The possibility remains that lifelong abstainers differ from drinkers in other traits potentially related to CAD risk, such as psychological characteristics, dietary habits, or physical exercise patterns. As previously stated, however, no substantial evidence exists indicating that such a trait occurs in persons of both sexes, various countries, and multiple racial groups. A causal, protective effect of alcohol is a simpler and more plausible explanation for the observed association.

## Relationship of Drinking to Total Mortality

As stated earlier, the relationship of moderate alcohol consumption to overall mortality in population studies is dominated by the lower CVD risk of moderate drinkers. Moderate drinkers are at slightly lower risk than abstainers and at considerably lower risk than heavier drinkers. Because of increased *non-CVD* mortality among heavier drinkers (e.g., from cancer, accidents, and cirrhosis), the *overall* alcohol-mortality risk curve is more or less J-shaped, rather than U-shaped, with heavier drinkers at highest risk, lighter drinkers at lowest risk, and abstainers at intermediate risk (Klatsky 1995*a*; Klatsky et al. 1992).

## Conclusion and Public Health Considerations

This brief survey documents the evidence for the disparity of alcohol’s effects on risk for different CVDs. Table 3 summarizes the relationships, with emphasis on the disparity between the overall favorable relations of lighter drinking and the overall unfavorable relations of heavier drinking.

Nonetheless, public health advice cannot be formulated easily. A more plausible option is for individual health practitioners, using personal knowledge of their clients, to privately advise those of their patients who are concerned about the probable health effects of drinking (Friedman and Klatsky 1993).

In general, heavier drinkers (i.e., men who consume three or more drinks per day and women who consume two or more drinks per day) are at increased risk of major social, personal, and adverse health consequences and should abstain from alcohol or reduce their level of consumption. In addition, because most nondrinkers have important personal or health reasons for abstention, *indiscriminate* advice to drink for health should not be given. Finally, the majority of the population consume only moderate amounts of alcohol and are therefore, as a group, at lowest total mortality risk. Persons at higher-than-average CAD risk who drink moderately should not be advised to reduce their alcohol consumption unless they are at special risk for disorders induced or aggravated by alcohol (e.g., liver disease or alcoholism).

## Figures and Tables

**Table 1:** Relative Risk of Death of Various Cardiovascular Conditions and Cirrhosis by Ex-Drinkers’ Former Level of Alcohol Use

Condition (number of deaths)	Relative Risk for Each Drinking Category (drinks per day)					

	Ex-drinkers	<1/mo	<1/day but >1/mo	1–2/day	3–5/day	6+/day
All coronary artery disease (CAD) (600)	1.0	0.9	0.8^a^	0.7^a^	0.7^a^	0.8
Acute myocardial infarction (284)	1.0	0.7	0.8	0.6^a^	0.5^a^	0.6
Other CAD (316)	0.9	1.0	0.7	0.8	0.7	1.0
Stroke (138)	1.0	0.8	0.8	0.8	0.7	1.4
Hemorrhagic (41)	1.4	1.5	1.6	1.8	1.3	4.7
Ischemic (34)	0.9	0.5	0.5	0.3	0.4	—b
Nonspecific (63)	1.1	0.7	0.9	1.0	1.0	1.2
Hypertension (64)	2.8	2.4	1.9	1.3	2.2	2.1
Cardiomyopathy (24)	3.4	8.5^a^	4.0	5.6	2.4	8.0
Syndromes* (82)	0.6	0.6	0.5	0.4^a^	0.6	1.0
Arterial** (41)	—b	1.1	1.6	0.4^a^	1.7	—b
Cirrhosis (42)	10.8^a^	1.4	1.0	4.3	8.1^a^	22.0^a^

* Includes “symptomatic heart disease” (*n* = 32); disorders of heart rhythm (*n* = 22); and ill-defined heart disease (*n* = 28).

** Includes arteriosclerosis (*n* = 15); aneurysms (*n* = 23); peripheral vascular disease (*n* = 2); and arterial embolism and thrombosis (*n* = 1).

a Significantly different from 1.0.

b Insufficient cases for estimate.

NOTE: Relative risk is defined as the CAD death risk for each drinking category compared with the CAD risk among lifelong abstainers. This comparison is expressed as a ratio, using the CAD risk among abstainers as a reference, set at 1.0. For example, people who consume one to two drinks per day are almost twice as likely to die from hemorrhagic stroke than are abstainers (i.e., relative risk = 1.8), but they are approximately one-half as likely to die from acute myocardial infarction than are abstainers (i.e., relative risk = 0.6). Adjustments have been made for the influence of gender, age, race, smoking, education, and coffee consumption.

SOURCE: Adapted from Klatsky et al. 1990*a*.

**Table 2:** Effect of Beverage Choice on Relative Risk for Coronary Artery Disease (CAD)

Group	Wine	Relative Risk for CAD	

		Liquor	Beer
Both sexes	0.8^a^	0.9^a^	0.7^a^
Men	0.9	0.9	0.7^a^
Women	0.7^a^	0.9	0.7

a RR significantly different from 1.0.

NOTE: Relative risk (RR) is defined as the CAD death risk for each drinking category compared with the CAD risk among lifelong abstainers, adjusted for total alcohol consumption. For example, among both sexes, although all beverage types afford some protection against CAD (i.e., RR is less than 1.0 in each case), RR is lowest among beer drinkers and highest among those who consume liquor. This comparison is expressed as a ratio, using the CAD risk among abstainers as a reference, set at 1.0. Results are adjusted for age, sex, race, smoking, education, marital status, and obesity.

SOURCE: Adapted from Klatsky et al. 1997.

**Table 3:** Relationships of Alcohol Consumption to Cardiovascular Diseases (CVDs)

Amount of Alcohol Drinking*			
Condition	Moderate	Heavy	Comment
Dilated cardiomyopathy	No relationship	Probably causal in some cases	Unknown cofactors may influence association
Hypertension (HTN)	Little/no relationship	Probably causal	Mechanism unknown
Coronary artery disease (CAD)	Protective	Possibly protective	Dominates *all* epidemiologic data on CVD
Arrhythmia	Probably none	Probably causal in some cases	Other alcohol-related CVDs may influence susceptibility
Hemorrhagic stroke	May increase risk	Increased risk	Via HTN and inhibition of blood clotting
Ischemic stroke	Protective	Possibly protective	Complex interactions with other CVDs and risks

* Moderate drinking = consumption of fewer than three standard drinks per day; heavy drinking = consumption of three or more standard drinks per day. A standard drink = approximately 12 ounces of beer, 5 ounces of wine, or 1.5 ounces of distilled spirits, each of which contains approximately 12 grams (0.5 ounce) of alcohol.

## Footnotes

1 See glossary, p. 23, for terms not defined in the text.

2 Relative risk is defined as the CAD death risk for each drinking category compared with the CAD risk of lifelong abstainers.

3 Conversely, low-density lipoprotein (LDL) cholesterol is a blood constituent that contributes to the deposition of fatty deposits within the coronary arteries.

4 These different types of protective substances have been reported mostly in red wine, although low concentrations of polyphenols exist in certain white wines and beer.

## GLOSSARY

Aneurysm: A dilated portion of an artery, probably caused by a structural weakness in the arterial wall and commonly associated with *atherosclerosis.* Although some aneurysms are without symptoms, they may promote the formation of *emboli* and are subject to sudden rupture.
Angina pectoris: A manifestation of coronary artery disease (CAD), characterized by poorly defined chest discomfort.
Arrhythmia: Irregular heartbeat.
Atherosclerosis: Thickening and hardening of the coronary arteries, caused largely by the deposition of fatty substances.
Cardiomyopathy: Any disease of heart muscle, often characterized by enlargement and flabbiness of the heart muscle, leading to fatigability, shortness of breath, and progressing to *congestive heart failure*.
Congestive heart failure: Inadequacy of heart function, often characterized by shortness of breath, fluid accumulation in tissues, and complications including *arrhythmia, embolism,* and sudden death.
Coronary artery disease (CAD): Narrowing of the coronary arteries resulting from *atherosclerosis,* which promotes the formation of blood clots that may obstruct the flow of blood in the arteries, depriving heart muscle tissue of its oxygen supply. The major manifestations of CAD are *angina,* myocardial *ischemia,* and sudden death.
Dilated cardiomyopathy: Any cardiomyopathy in which one or more chambers of the heart are abnormally distended with blood. This category of conditions includes alcoholic cardiomyopathy.
Embolism: Blood clots or fatty substances that travel through the bloodstream. Emboli that lodge in blood vessels of the brain can cause *ischemic stroke.*
Hemorrhagic stroke: A condition in which a ruptured artery interrupts the brain’s blood supply, depriving the affected area of oxygen. The pressure of accumulating blood may also cause tissue swelling and damage. Symptoms may include sudden severe headache, vomiting and, ultimately, coma.
Hypertension: High blood pressure, frequently defined as pressure in excess of 140/90. The significance of a blood pressure measurement may depend on age, emotional state, or other factors.
Ischemia: Local oxygen deprivation within a tissue, generally resulting from blockage of a blood vessel.
Ischemic stroke: Brain damage caused by blockage of an artery supplying the brain. Symptoms of sensory, intellectual, or motor impairment may appear suddenly, often resulting in death or permanent damage.
Myocardial infarction: A “ heart attack” ; a manifestation of *CAD* in which oxygen deprivation leads to death of heart muscle cells in the affected region, followed by scar formation that may continue to impair heart function. The condition often leads to death, instantly or later, sometimes from another heart attack, or from complications such as *arrhythmias,* heart failure, or *embolism.*
Peripheral vascular disease: Any disorder of the circulatory system that does not directly involve the heart or coronary arteries. The term is often taken to exclude strokes as well.
